# 

**Published:** 1878-03

**Authors:** 


### CONTENTS.

Histology and Microscopy.—*By W. H. Atkinson, M. D, D. D. S*.... 89
Draft of a Bill to Amend the law relating to Dental Practitioners in Great Britain........ 107

PROCEEDINGS OF SOCIETIES.

Annual Meeting of the Brooklyn Dental Society........ 119

SELECTIONS.

Dangerous Colors in Wall Papers........ 125
Cyanide of Zinc in Facial Neuralgia........ 125
A New Theory of the Nature of Water........ 125
Opium........ 125

EDITORIAL.

A Bill relating to Dental Practitioners in Great Britain........ 126
Michigan Dental Association........ 127
Dental Law........ 129
Cincinnati Bi-Ennial Musical Festival........ 129
A New Medical Journal........ 130
The Dental and Oral science Magazine........ 131
Vick's Illustrated Monthly Magazine........ 131

